# A paradigm shift in public health structures: creating sustainable systems to achieve global healthcare equity

**DOI:** 10.1186/2047-2994-4-S1-P105

**Published:** 2015-06-16

**Authors:** D Schafer, A Moten

**Affiliations:** 1 McDaniel College, Westminster, MD, USA; 2 Healthnovations International, Houston, TX, USA; 3 National Capital Consortium, Uniformed Services University of the Health Sciences, Bethesda, MD, USA

## Introduction

A paradigm shift is required to effectively serve the public health needs of the developing world. Such a shift will rely on more efficient partnerships between public health entities, as well as more sustainable models of providing care, and the application of new medical technologies.

## Objectives

Low- and middle-income countries currently face a public health epidemic due to the inability to properly serve resource-poor and remote communities. Lack of communication between the many nonprofit, governmental, and other entities involved plague attempts to increase health of resource-poor populations. An organized approach that includes local, national, and international agents is required to efficiently provide public health services. Such a paradigm shift would be sustainable, unlike current models, and allow local medical professionals to take charge in the administration of care with greater effectiveness.

## Methods

By aligning efforts of governments, nonprofit organizations, and other entities, more efficient and effective care can be administered to those who need it. We propose to organize these efforts, while also teaching and equipping healthcare professionals in developing settings to run self-sufficient and sustainable clinics for the rural poor. Along with building local health infrastructure and establishing partnerships, research and implementation of novel therapeutics must be explored.

## Results

Sustainable healthcare administration at the local level is essential. Healthnovations International works to empower local medical professionals to provide sustainable care for those in resource-poor settings. Local clinics are provided with equipment and other necessities to help them become self-reliant. Once this independence is achieved, the local health professionals working in the clinic take on full administrative responsibility.

## Conclusion

By encouraging thoughtfully orchestrated efforts across governmental and not-for-profit entities, resources can be intelligently distributed to those who need them most. Meanwhile, to complement this top-down approach to global health, local clinics and village medical professionals must be empowered to deliver care both effectively and sustainably. By balancing these two approaches, true health equity can be achieved around the world.

## Disclosure of interest

None declared.

